# p-Type Organic Semiconductor–Metal Nanoparticle Hybrid Film for the Enhancement of Raman and Fluorescence Detection

**DOI:** 10.1021/acs.jpcc.4c08030

**Published:** 2025-02-12

**Authors:** Rongcheng Gan, Dominik Duleba, Robert P. Johnson, James H. Rice

**Affiliations:** 1 School of Physics, University College Dublin, Belfield, Dublin 4 D04P7W1, Ireland; 2 School of Chemistry, University College Dublin, Belfield, Dublin 4 D04 V1W8, Ireland

## Abstract

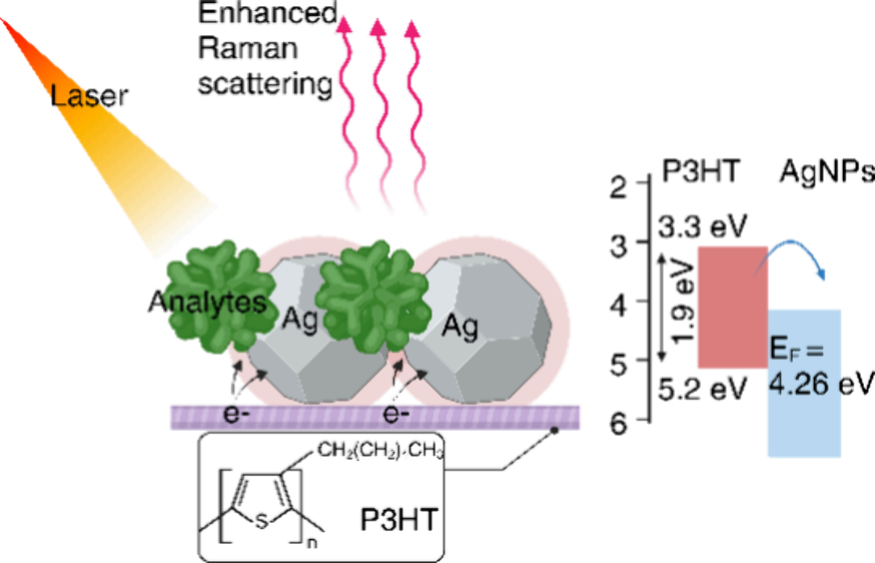

Hybrid platforms of organic semiconductors and plasmonic metal nanostructures have the potential to form effective optical detection substrates. Here, we report the use of an organic p-type conducting polymer poly(3-hexylthiophene-2,5-diyl) combined with plasmon-active silver nanostructures to enhance both Raman and fluorescence signal intensities. This enhancement occurs when optically excited charge from the polymer is transferred to silver, causing an enhancement of the electromagnetic field and leading to an increase in both the Raman and fluorescence signal intensities. This study demonstrates the potential of the organic semiconducting polymer–plasmonic metal nanostructure platform in spectroscopy detection technology.

## Introduction

Surface enhanced Raman spectroscopy (SERS) is a highly sensitive and nondestructive vibrational spectroscopy technique used to detect chemical and biological molecules at low concentrations^1−3^ with applications across medicine, biology, chemistry, environmental science, and security.^4−8^ Traditionally, SERS involves adsorbing analyte molecules on noble-metal substrates, such as gold or silver.^9−11^ The enhancement of Raman scattering intensity in SERS arises from localized surface plasmon resonance (LSPR) excitation (electromagnetic mechanism) and chemical interaction (chemical mechanism),^12−14^ which provides a Raman enhancement factor (EF) of around 10^6^^15^ and 10^3^,^16^ respectively.

Semiconductors, such as biomaterials^17−19^ or metal oxides, have been explored as alternatives to metal SERS substrates.^20,21^ Notable materials like ZnO,^22^ TiO_2_,^23^ InAs/GaAs,^24^ and LiNbO_3_^25^ as well as novel semiconductors such as Ta_2_O_5_^26^ and W_18_O_49_,^27^ have shown potential as effective SERS platforms. Semiconductors are advantageous because they can be cost-effective, easily processed, and biocompatible.^28,29^ However, the enhancement factors and detection limits of semiconductor SERS substrates are generally lower compared to metal substrates.^30^ To address this, introducing point defects such as vacancies,^31^ atom substitutions,^32^ and interstitial atoms^33^ in the crystal lattice of semiconductors has been proposed. These defects can create additional energy levels, allowing for optical gap tuning and improving the effectiveness of the charge transfer mechanism for specific probe molecules.^34^ One common method is the introduction of oxygen vacancies (VO) in the semiconductor structure.^22,35^ Studies have shown that surface enhancement of Raman scattering (SERS) can be achieved by incorporating photoinduced surface oxygen vacancy states on semiconductors such as TiO_2_ surfaces. These oxygen vacancy defects facilitate semiconductor–defect–metal–analyte vibronic couplings, which enhance the Raman signal.^36^

Adding a metal to the semiconductor can form metal–semiconductor junctions that enable effective carrier separation through Schottky junctions.^37^ These junctions form when metal and semiconductor are in close contact, causing electrons to migrate between the two materials to equilibrate their Fermi levels.^38^ Here, we examine the potential of combining a p-type conducting polymer (poly(3-hexylthiophene (P3HT)) with plasmon active metal nanoparticles. Poly(3-hexylthiophene (P3HT) is a narrow band gap polymer (*E*_g_ = 1.9 to 2.1 eV conducting polymer).^39−41^ Compared to other organic semiconductor thin films, such as graphene, which require high temperatures and complex preparation procedures, P3HT thin films have a simple and time-efficient fabrication process.^42,43^ P3HT is a narrow band gap semiconductor, which enables the Raman excitation laser to create interband electronic transitions.^44^ Wider band gap semiconductors such as cellulose or diphenylalanine and their derivatives require the use of UV light to create interband electronic transition.^45−49^ P3HT forms a Schottky junction with metals like silver, creating ohmic contacts for extracting charges enabling effective charge transfer between the silver and the semiconductor.^50^

**Figure 1:**
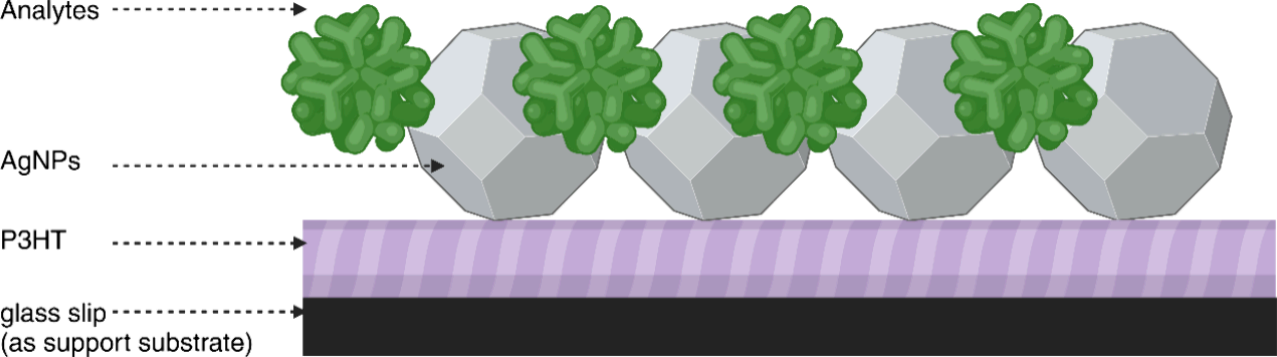
Illustration of the P3HT/AgNP substrate.

We study a bilayer material formed from a thin film of P3HT prepared with a layer of silver nanoparticles (AgNPs) (Figure 1). We demonstrate that this semiconductor polymer–plasmonic metal hybrid platform effectively increases the Raman scattering and fluorescence intensity with a series of probe molecules achieved through efficient charge transfer from the optically excited polymer to the silver enhancing the metals electromagnetic field strength, occurring as the electron charge is not efficiently transported in the p-type semiconductor, promoting transfer to the silver. Additionally, P3HT is hydrophobic with contact angles with water typically in the range of 90° to 100°,^51^ which supports the substrate's ability to enhance Raman or fluorescence detection.^52,53^

## Methods

### Preparation of Chemical Solution

P3HT (445703, Sigma-Aldrich) is dissolved in CHCl_3_ (Fisher Scientific) to a concentration of 1 mg/mL. For Raman measurements, methylene blue (MB) (M9140, Sigma-Aldrich) powder was dissolved in deionized water to an initial concentration of 10^–3^ M and further diluted to 10^–5^ M. The same preparation procedure was used with meso-tetra(*N*-methyl-4-pyridyl)porphine tetrachloride (TMPyP) (T40125, Frontier Scientific), Rhodamine 6G (R6G) (252433, Sigma-Aldrich), and Rhodamine (RhB) (Acros Organics). For fluorescence measurements, 500 mg of poly(vinyl alcohol) (PVA) (10854, Fluka) was dissolved in 10 mL of deionized water to a concentration of 50 mg/mL. Next, RhB solution was mixed with PVA solution at a 1:5 ratio.

### Preparation of SER Chips

Coverslips were cleaned with deionized water and dried with N_2_. Next, 200 μL of P3HT solution was spin-coated on coverslips using a homemade spin-coat device. A thin layer of P3HT formed after the CHCl_3_ evaporated. Then, 50 μL of AgNPs (730807, Sigma-Aldrich) was drop-cast on top of the P3HT thin film and coverslips.

### UV–Vis Absorbance

Optical absorbance data were measured with a Bruker Tensor 27 with a P3HT thin film on glass coverslips and a solution of AgNPs (0.02 mg/mL) and probe molecules (10^–5^ M) on a quartz cuvette. The measurement setup is follows: 1 nm step size, bandwidth of 5 nm, and data range of 400–4000 cm^–1^.

### Fourier Transform Infrared Spectroscopy (FTIR)

An Alpha Platinum Bruker system was used for collecting FTIR data of P3HT thin film on glass coverslips. The measurement setup as follows: resolution of 4 cm^–1^, sample scan time of 32 scans, and 300–999 nm wavelength range.

### Scanning Electron Microscopy (SEM)

Scanning electron microscopy images were captured by a Hitachi Regulus 8230 system with a P3HT:AgNP substrate.

### Raman Spectroscopy

Raman spectra were captured by a system, composed of 532 nm monochromatic laser power supplies, an Olympus IX71 inverted optical microscope, a spectrograph (Andor Kymera 328i), and an Andor iXon Ultra 897. Beamsplitters and long pass filters (Shamrock) were equipped on the microscope’s filter wheel for all excitation lasers. The measurement used a ×20 objective to focus the laser on the sample and an exposure time of 1 s. Raman spectra were taken as the mean data of three measurements.

### Fluorescence Spectroscopy

The fluorescence data was obtained utilizing the same equipment as described for Raman spectroscopy. The exposure time was 0.1 s, and fluorescence spectra were taken as the mean data of three measurements.

## Results and Discussion

**Figure 2:**
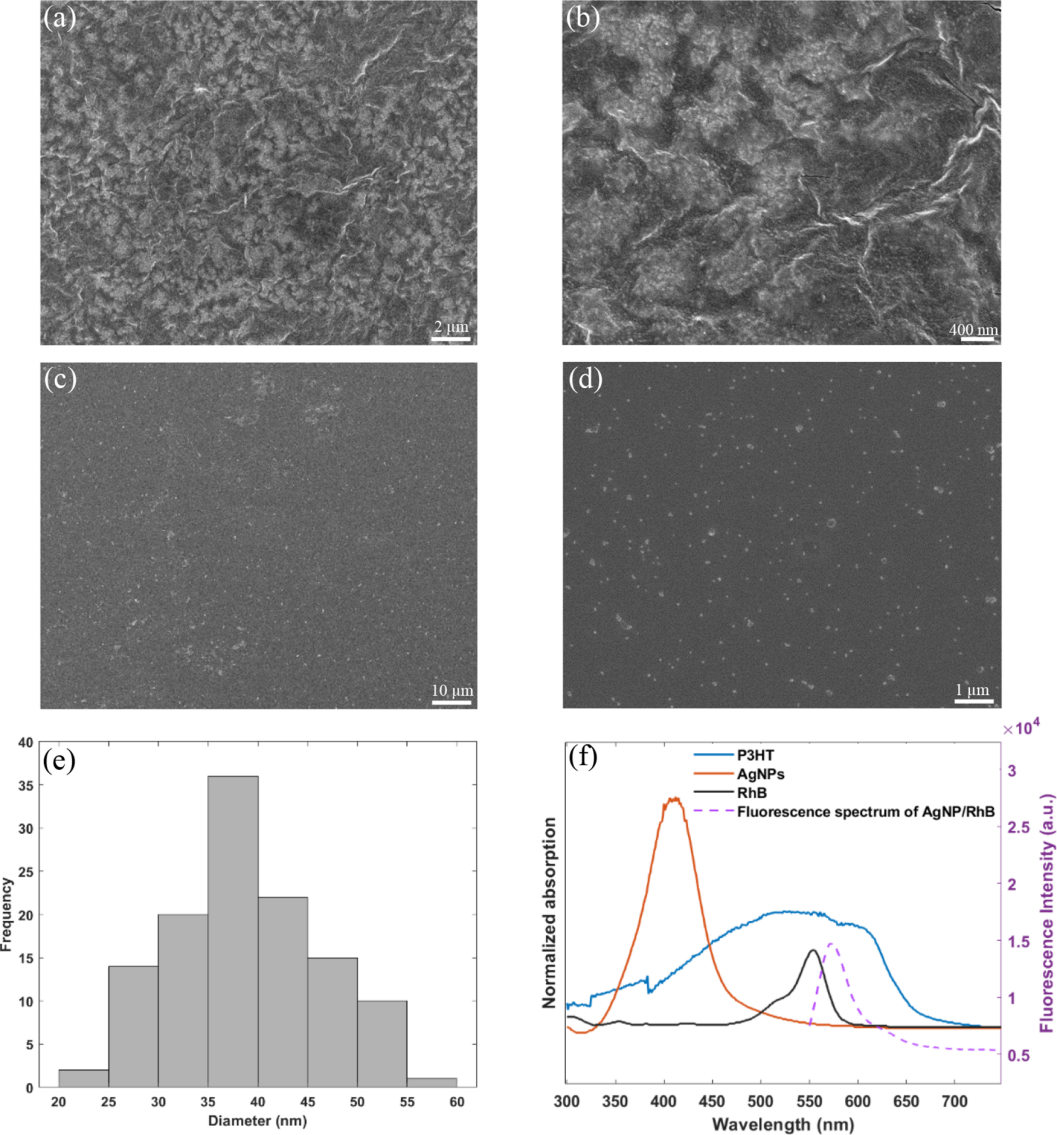
Scanning electron microscopy (SEM) image of (a, b) P3HT and (c, d) AgNPs in the surface of P3HT; (e) histogram plot of nanoparticle size vs occurrence. Showing the average nanoparticle size is 40 nm; (f) optical absorption spectra of P3HT (blue), AgNPs (orange), and Rhodamine B and fluorescence spectra recorded for Rhodamine B embedded in polymer matrix AgNPs (purple).

The substrate was prepared by depositing AgNPs onto P3HT. Scanning electron microscopy (SEM) imaging was undertaken to characterize the substrate (Figure 2(a–e), Supporting Information Figure S1). SEM imaging revealed the structure of P3HT thin film (Figure 2(a,b)) and the AgNP clusters distributed evenly on the P3HT substrate (Figure 2(c,d)). Analysis of the SEM images showed that the average size of AgNPs is 40 nm (Figure 2(e), Figure S1). Fast Fourier transform infrared (FTIR) and optical absorption spectroscopies were applied for additional characterization of the sample. The FTIR spectrum of P3HT shows a series of peaks in the range of 800–3000 cm^–1^ that agree with literature reports.^54−56^ The optical absorption spectrum (Figure 2(f)) of AgNPs shows a broad band at around 430 nm arising from the localized surface plasmon resonance (LSPR) of the nanoparticles. This matches the LSPR frequency reported for 40 nm AgNPs.^29^ For P3HT thin film, the absorption spectrum (Figure 2(f)) shows a broad peak between 450 and 650 nm.^30^ The onset of absorption is proportional to the optical bandgap of 650 nm (1.9 eV). The broad absorption peak were from π–π* transitions within the conjugated polymer backbone (500–550 nm (2.2–2.5 eV)) and exciton formation and the lower energy tail of the π–π* transition (600–700 nm (1.9–2.1 eV)).^30^

**Figure 3:**
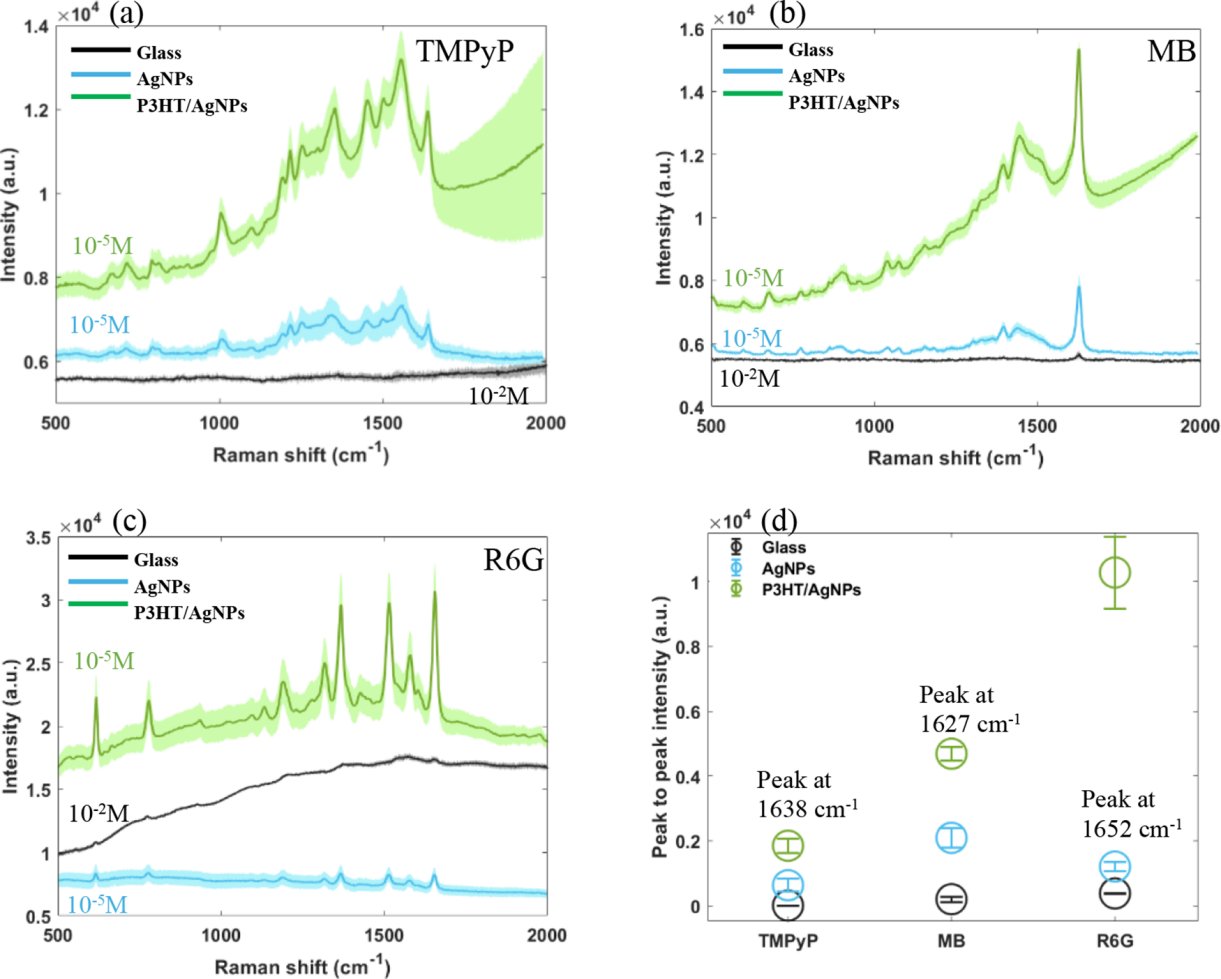
SERS studies undertaken using a Raman excitation wavelength of 532 nm: (a–c) SERS spectra recorded on P3HT/AgNPs (green), glass/AgNPs (blue), and glass (black) for TMPyP, R6G and MB. (d) Plot of peak-to-peak intensity for each probe molecule on P3HT/AgNPs (green), glass/AgNPs (blue), and glass (black).

We investigated the impact of the developed substrate on SERS signal intensity. To do this, a series of probe molecules were prepared on P3HT/AgNPs, glass/AgNPs, and glass substrates. Specifically probe molecules meso-tetra(*N*-methyl-4-pyridyl)porphine tetrachloride (TMPyP), methylene blue (MB), and Rhodamine 6G (R6G) were studied (Figure S3, Supporting Information). The SERS spectra for each probe molecule were recorded (Figure 3).

The SERS spectra of TMPyP show a number of peaks such as those at 1638, 1555, and 1253 cm^–1^, which relate to pyrrole bending, C–C stretching, and C–pyrrole bending.^57^ MB’s Raman spectrum shows peaks at 1445 cm^–1^ (C–N stretching) and 1627 cm^–1^ (aromatic C–C stretching).^58^ The Raman spectrum of R6G shows peaks at 615 cm^–1^ related to the C–C–C ring in-plane vibration, 775 cm^–1^ related to C–H out-of-ring deformation, and 1652 cm^–1^ related to C–C stretching.^59^ For all three probe molecules, the SERS spectra recorded on P3HT/AgNPs matched literature reports.^49,58,59^

Shown also in Figure 3(a–c) are the SERS spectra for each probe molecule recorded on glass/AgNPs as well as on P3HT/AgNPs. The overall SERS signal intensity of each probe molecule was stronger on P3HT/AgNPs than on glass/AgNPs. Comparing the Raman peak-to-peak ratio for the SERS spectra shows a 2-fold signal increase for MB and TMPyP and an 8-fold signal increase for R6G (Figure 3(d)) when using P3HT/AgNPs as compared to glass/AgNPs.

The Raman enhancement factor was calculated by comparing the ratio of the Raman signal intensity from the probe molecule adsorbed on a P3HT/AgNPs to the Raman signal intensity from the same molecule on glass:
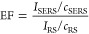
where *I*_SERS_ is the intensity of the Raman signal from the molecule on P3HT/AgNPs, *I*_RS_ is the intensity of the Raman signal from the same molecule on glass, *c*_SERS_ is the concentration of probe molecules on P3HT/AgNPs, and *c*_RS_ is the concentration of probe molecules on glass. The EFs for MB (peak at 1627 cm^–1^) and R6G (peak at 1652 cm^–1^) on P3HT/AgNP substrate are 2.40 × 10^4^ and 2.76 × 10^4^, respectively.^60^

The SERS enhancement seen for the P3HT/AgNP substrate can potentially arise through a combination of the electromagnetic mechanism (EM) and the chemical mechanism (CM). The EM mechanism enhances SERS as a result of the magnification of the local electromagnetic field in both the excitation and scattering steps due to the excitation of local surface plasmon resonance at the metal surface. CM is typically based on photoinduced charge transfer (PICT) processes between absorbed molecules, semiconductors, and/or a metal surface creating conditions for stronger SERS signal intensities.^61,62^ P3HT possess a band gap of 1.9 eV which can generate electron–hole pairs after irradiation with the Raman excitation laser (532 nm, 2.3 eV).^63^ The electrons in P3HT are not transported freely in the p-type polymer and can potentially undergo photoinduced charge transfer (PICT) to AgNPs where the Fermi level of the metal is lower than the valence band of P3HT. This extra charge on the AgNPs can create stronger EM fields resulting in SERS signal enhancement. Additionally, P3HT is hydrophobic (contact angle > 90°).^51^ This can support enhanced SERS signal intensities by preventing the analyte from spreading or diluting over the surface when it is added to the surface using water as the solvent.

To better understand the mechanism behind the observed results, we carried out finite element analysis using COMSOL Multiphysics. The electromagnetic enhancement can be calculated by modeling the propagations of the electromagnetic waves. The model consists of AgNPs with a 40 nm diameter placed on the 100 nm-thick P3HT thin film. The P3HT thin film is supported by a thick layer of glass, while above the film, the modeled material is air. The incoming electromagnetic wave is incident at a 90° angle onto the thin film, with the light polarized to the *x*-axis. Floquet periodicity is used along the *x*-axis. The optical properties of Ag are taken from Johnson and Christy,^64^ P3HT from Hussein et al.,^65^ and glass from Ghosh et al.^66^

The electromagnetic enhancement is calculated from the steady-state solution to a 532 nm incident beam. The electric field enhancement around the particle relative to a baseline is used to calculate the Raman enhancement factor:^67^
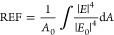


**Figure 4:**
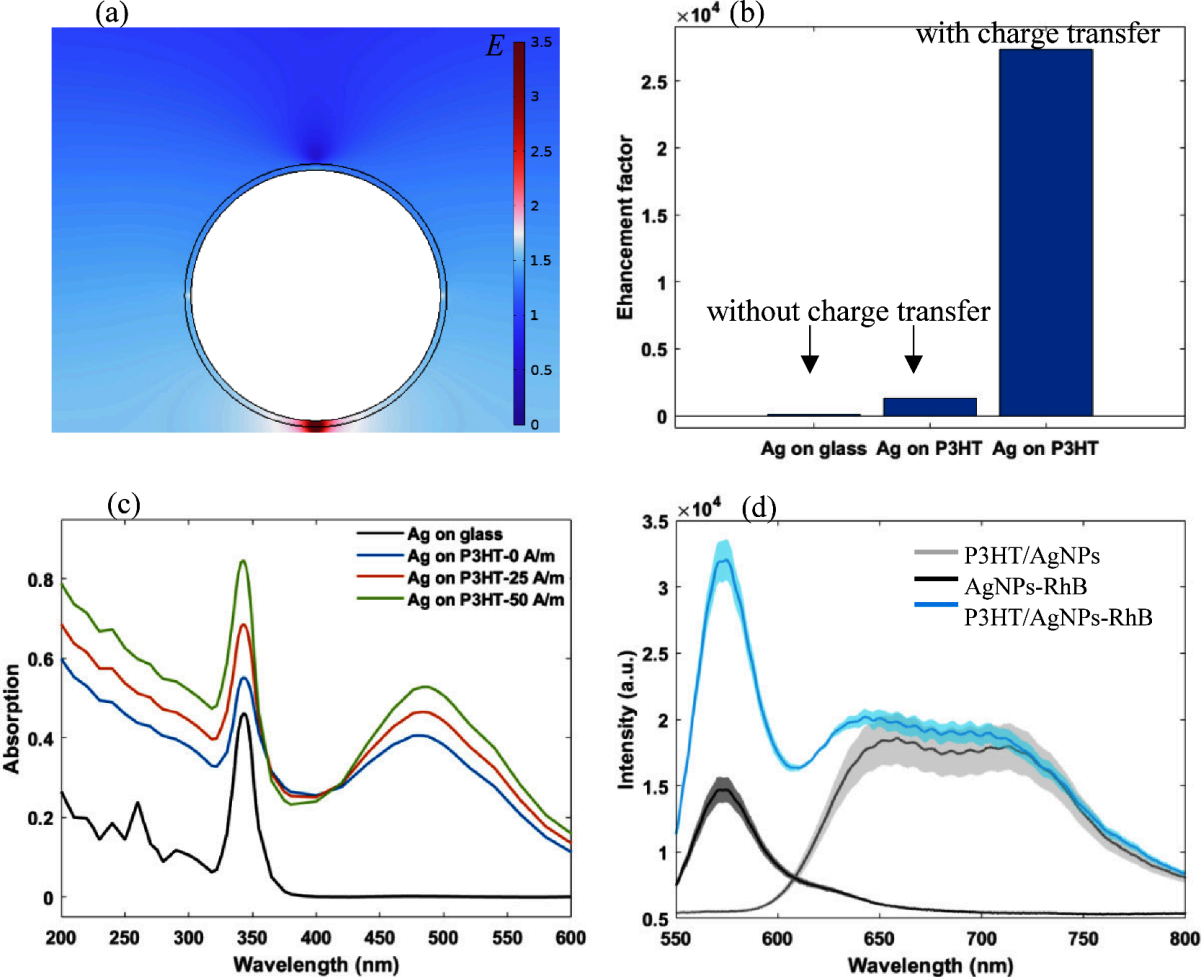
COMSOL Multiphysics simulation of (a) electromagnetic field distribution of P3HT/AgNP substrate; (b) enhancement factor from Ag on glass, Ag on P3HT (without charge transfer), and Ag on P3HT (with charge transfer); (c) optical absorption spectra of Ag on glass (black) and P3HT with charge transfer from 0 A/m (blue), 25 A/m (orange), and 50 A/m (green); (d) fluorescence spectra recorded for Rhodamine B embedded in a polymer matrix on P3HT/AgNPs (blue) and glass/AgNPs (black). Shown also is the florescence spectrum recorded for P3HT/AgNPs (gray) without dye molecules added.

The enhancement contributed by each component is calculated in a stepwise manner (glass surface vs Ag on glass, Ag on glass vs Ag on P3HT, Ag on P3HT vs Ag on P3HT with charge transfer). Charge transfer is qualitatively approximated by applying a surface current density to the surface of the P3HT as well as to the bottom surface of the particle. As shown on Figure 4(a,b), the charge transfer between P3HT and AgNPs contributes an effective enhancement for the electromagnetic field at the interface of the AgNP and P3HT substrate, resulting in a Raman enhancement factor that is strongly increased on the P3HT/AgNP substrate. In addition, the P3HT/AgNP substrate has an extra absorption peak around 500 nm and can be increased by charge transfer from P3HT to Ag (Figure 4(c)). The simulation results indicated that photoinduced charge transfer from P3HT to the silver nanostructure effectively enhanced the electromagnetic field surrounding the silver nanoparticle. Thus, it theoretically confirmed the contribution of electromagnetic enhancement mechanism for Raman enhancement.

We also investigated the performance of the P3HT/AgNP substrate in fluorescence enhancement. As simulations (Figure 4(a–c)) show that EM mechanism is responsible for SERS enhancement, fluorescence enhancement can also potentially occur. As reported, local surface plasmon resonance in metallic nanostructures increases the fluorophore’s excitation rate by amplifying the local electromagnetic field near the surface and boosting the radiative decay rate of fluorophores, leading to enhanced fluorescence intensity.^68^ When a fluorophore is in close proximity to a metal surface (<5 nm), its excited-state energy transfers to the electron–hole pairs of the metal, dissipating in the metal instead of fluorescence.^69,70^ To avoid fluorescence quenching, a dielectric polymer was applied to introduce nanoscale separation (>5 nm) between the fluorophores and metal surface.^68^ We prepared RhB (concentration of 10^–5^ M) mixed with poly(vinyl alcohol) (PVA), which was drop-cast onto the substrates P3HT/AgNPs and glass/AgNPs before fluorescence measurement.

The fluorescence spectra (Figure 4(d)) show a fluorescence peak of RhB located at around 575 nm. A 2-fold enhancement of fluorescent signal can be observed when using P3HT/AgNPs compared to a glass/AgNP substrate. The peak around 650 nm is seen when using the P3HT/AgNPs substrate (Figure 4(d)), which is absent when glass/AgNPs is used. This peak is assigned to emission from P3HT.^71^ This enhancement in fluorescence using P3HT/AgNPs potentially arises from the same mechanism as that in SERS (as outlined above) where PICT to AgNPs following 532 nm excitation creates an additional charge on the AgNPs. This results in stronger EM fields, creating stronger SERS signal enhancement.

## Conclusions

Hybrid platforms of organic semiconductors and plasmonic metal nanostructures have the potential to form effective optical detection platforms. Here, we report the use of an organic p-type conducting polymer poly(3-hexylthiophene-2,5-diyl) (P3HT) combined with plasmon-active silver nanostructures to enhance both Raman and fluorescence signal intensities. This enhancement occurs when optically excited charge from P3HT is transferred to silver, causing an enhancement of the electromagnetic field and leading to an increase in both Raman and fluorescence signal intensities. The enhancement mechanism was theoretically verified by COMSOL Multiphysics simulation. This study demonstrates the potential of the organic semiconducting polymer–plasmonic metal nanostructure platform as a spectroscopy detection technology.

## Supporting Information Available

SEM image of AgNPs on P3HT, FT-IR data, optical absorption spectra (PDF)
