# Supplementary material for: p-Type Organic Semiconductor–Metal Nanoparticle Hybrid Film for the Enhancement of Raman and Fluorescence Detection
Source: J Phys Chem C Nanomater Interfaces. 2025 Feb 12;129(7):3659–66. doi: 10.1021/acs.jpcc.4c08030 (PMC11848917; doi:10.1021/acs.jpcc.4c08030)
Supplement: Supplementary file 1 — jp4c08030_si_001.pdf [file jp4c08030_si_001.pdf]

P-type Organic Semiconductor – Metal Nanoparticle Hybrid Film for the Enhancement of Raman and Fluorescence Detection

Rongcheng Gan [1], Dominik Duleba[2], Robert Johnson[2], James Rice [1*]

[1]School of Physics, University College Dublin, Belfield, Dublin 4, D04P7W1, Ireland.

[2]School of Chemistry, University College Dublin, Belfield, Dublin 4, D04V1W8, Ireland.

*correspondence: james.rice@ucd.ie

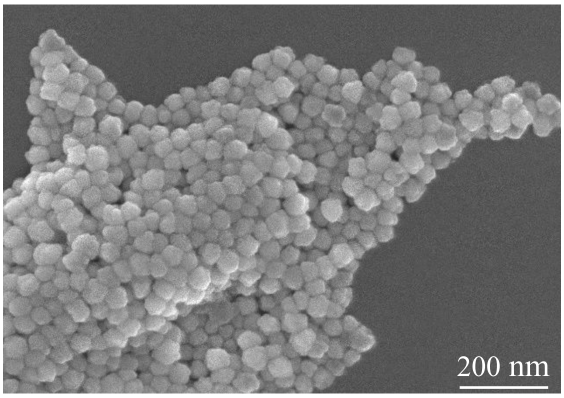

Figure S1. SEM image of AgNPs in the surface of P3HT.

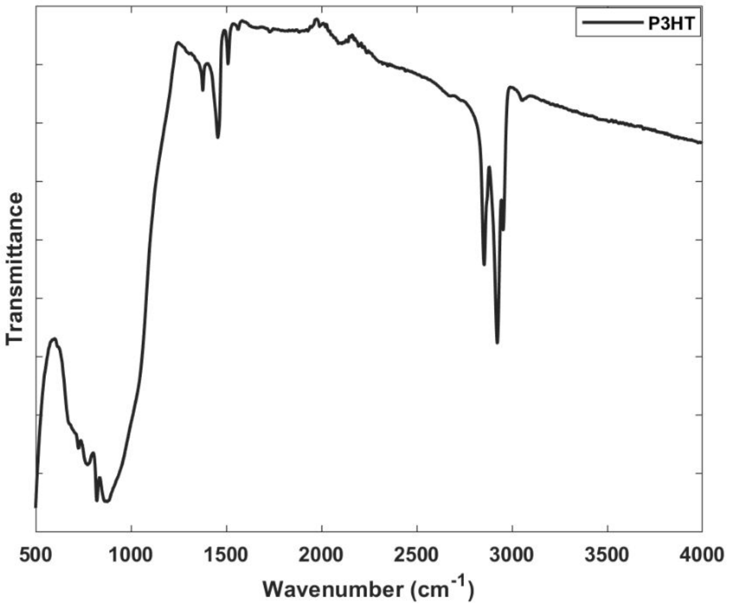

Figure S2. FTIR spectrum of P3HT

Fast transform infrared transmission (FTIR) spectra of P3HT shows a series of peaks in the range of 800 – 1000 cm^{-1} which recognized as thiophene ring, corresponding to C - S bond vibrations, (2) 1400 – 1600 cm^{-1} for polymer chain, contributing by C = C stretching vibration and (3) 2600 – 3000 cm^{-1} for alkyl chain which assigned to C - H symmetric and asymmetric stretching vibration.

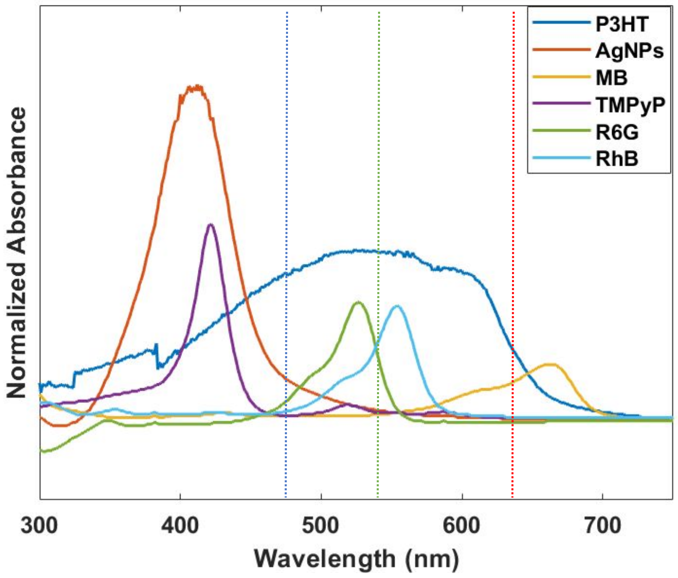

Figure S3. Optical absorption spectra for a series of molecules, which were used as probe molecules for SERS and fluorescence studies. Probe molecules meso-tetra(N-methyl-4-pyridyl)porphine tetrachloride (TMPyP), Methylene blue (MB) and Rhodamine 6G (R6G) where studied. The absorption spectrum for each of these molecules are shown with the spectra recorded for AgNPs and P3HT.
